# Allelopathic Trade-Offs of Rye and Wheat Residues Versus 2-Benzoxazolinone: Impacts on Cotton Growth

**DOI:** 10.3390/biology14101321

**Published:** 2025-09-25

**Authors:** Yue Li, Vivien G. Allen, Junping Chen, David B. Wester

**Affiliations:** 1 Agronomy College, Gansu Agricultural University, Lanzhou 730070, China; 2 State Key Laboratory of Aridland Crop Science, Lanzhou 730070, China; 3 Department of Plant and Soil Science, Texas Tech University, Lubbock, TX 79409, USA; 4 Plant Stress and Germplasm Development Unit, United States Department of Agriculture-Agriculture Research Service, Lubbock, TX 79401, USA; junping.chen@ars.usda.gov; 5 Caesar Kleberg Wildlife Research Institute, Texas A&M University-Kingsville, Kingsville, TX 78363, USA; david.wester@tamuk.edu

**Keywords:** allelopathy, 2-benzoxazolinone (BOA), cover crops, cotton, hormesis, residue biomass thresholds, residue persistence

## Abstract

Cover crops provide soil cover benefits but can impose allelopathic risks on cotton. We evaluated the functional trade-offs of rye and wheat residues versus purified 2-benzoxazolinone (BOA) under greenhouse conditions. Four experiments applied graded residue or BOA inputs in Pullman clay loam; cotton germination, height, chlorophyll (SPAD), and biomass were measured, and soil BOA, DIBOA, and DIMBOA were quantified by HPLC at designated sampling dates. Responses were dose dependent: BOA reduced germination linearly (−16.5% at 1000 nmol g^−1^ versus control) and shortened plants, and biomass and SPAD were directionally lower, most evident at 500 nmol g^−1^, but not statistically significant. Rye showed hormesis at 6400 kg ha^−1^ (+7.3% germination) and strong inhibition at 12,800 kg ha^−1^ (−31% germination; biomass up to −45%). Wheat produced intermediate inhibition (biomass −23.7%) and did not affect germination. In soil, benzoxazinoids exhibited significant rate effects at specific sampling dates followed by rapid decline. After storage, BOA showed no residual effects, whereas prior rye still reduced height, SPAD, and biomass. Framed at the agroecosystem scale, maintaining residue biomass below inhibitory thresholds and adjusting termination-to-planting intervals to avoid the early post-termination period, together with species or cultivar choice, can reconcile soil cover services with reliable cotton establishment.

## Simple Summary

Cover crops such as rye and wheat are widely used to protect the soil, save water, and reduce weeds in farming systems. While these benefits are clear, the remains of these plants left in the soil can also release natural chemicals that may slow down the growth of the next crop. Cotton is an important crop grown after cover crops in many regions, and farmers are often concerned that too much residue could reduce cotton yield. In this study, we grew cotton in greenhouse conditions and compared the effects of rye and wheat residues with those of a purified natural chemical produced by these plants. We found that moderate amounts of rye sometimes improved cotton germination, but high levels of residue greatly reduced germination and growth. Wheat had weaker effects, and the purified chemical alone caused only short-term changes. Our results show that the main risk comes from the residues themselves rather than from the chemical alone. These findings are important for farmers because they provide practical advice on how much residue can be left in the soil and how long to wait before planting cotton. By managing residue levels and timing, farmers can enjoy the benefits of cover crops while avoiding risks to cotton production.

## 1. Introduction

Cover crops are a cornerstone of sustainable agriculture, providing multiple ecosystem services such as enhancing soil quality, suppressing weeds, and reducing erosion [1,2,3]. Cover crop-based cotton systems are widely practiced in the southern United States, particularly Texas [4], and have been shown to improve yield and weed suppression in water-limited systems globally [5,6].Among small-grain cover crops, rye (*Secale cereale* L.) and wheat (*Triticum aestivum* L.) are widely adopted owing to their high biomass production and strong weed-suppressive capacity [7,8,9,10,11]. Nevertheless, growing evidence suggests that residues of these cereals can also negatively affect subsequent crops through allelopathy [12,13,14,15], which generally refers to the production and release of plant-derived secondary metabolites that directly or indirectly influence the germination, establishment, and growth of neighboring plants [16,17]. In cereals, allelopathic activity is primarily associated with benzoxazinoids such as 2,4-Dihydroxy-1,4-benzoxazin-3-one (DIBOA), 2,4-Dihydroxy-7-methoxy-1,4-benzoxazin-3-one (DIMBOA), and their degradation product BOA [18,19], which are released into soil and can inhibit germination and early seedling growth of sensitive species [20,21,22]. Rice et al. [23] quantified BX concentrations in both roots and shoots of rye at multiple growth stages, finding that BX glycosides dominate in root tissues and can rapidly convert into active aglycones when released into soil.

Cotton (*Gossypium hirsutum* L.) is a major cash crop in semi-arid regions such as the U.S. Southern High Plains, where conservation tillage and cover cropping are widely promoted to conserve soil and water resources [24,25]. Yet, the compatibility of cereal cover crops with cotton remains uncertain. Field studies have reported reduced cotton growth and yield following rye or wheat cover crops [26,27]. Although allelopathy is strongly implicated in these outcomes, the persistence of these effects and the relative contributions of purified allelochemicals versus residue-derived mixtures remain unresolved.

Benzoxazinoids degrade rapidly in soil, often within hours to days [28,29]. Nevertheless, inhibitory effects from cereal residues can persist into subsequent cropping cycles [30], suggesting that degradation products or interactions with soil microbes may prolong suppression [31,32]. Moreover, the extent of inhibition is dose-dependent: moderate residue inputs may stimulate germination through hormesis, whereas higher amounts typically result in strong suppression [33,34]. This dose-dependent and species-specific variability complicates the management of cereal–cotton rotations, where both soil conservation and crop productivity must be balanced. Despite extensive research on benzoxazinoid chemistry and field observations of rye allelopathy, few studies have systematically compared the relative effects of whole rye residues and purified BOA under controlled greenhouse conditions; such comparisons are essential for disentangling residue-mediated influences from the single-compound effects of BOA and for clarifying the mechanisms underlying cotton suppression. Framing residue use as allelopathic trade-offs—balancing soil cover services against carryover inhibition—enables evaluation of agroecosystem functional value, proxied here by establishment reliability and early-season biomass, under realistic management scenarios (residue load, species choice, and planting timing). This framing links benzoxazinoid pulses and persistence to decision-relevant levers—residue load (Experiment 1), the post-termination monitoring window (Experiment 2), delayed-planting carryover risk (Experiment 3), and species/compound choice (Experiment 4)—for improving establishment reliability in cover crop–cotton systems.

To address these uncertainties, we conducted four greenhouse experiments to investigate allelopathic suppression of cotton by rye, wheat, and BOA. The objectives were to: (i) quantify dose-dependent effects on cotton germination, biomass, and leaf chlorophyll content; (ii) track the dynamics of BOA and its derivatives in soil; and (iii) evaluate the persistence of allelopathic suppression over extended periods. By linking residue decomposition with plant responses, this study provides mechanistic insights and practical guidance for designing cereal cover crop–cotton systems.

## 2. Materials and Methods

### 2.1. Experimental Overview

Four greenhouse experiments were conducted over two consecutive years at Texas Tech University, Lubbock, TX, USA (33.58° N, 101.88° W) to investigate the allelopathic effects of cereal cover crop residues and a purified allelochemical on cotton (*Gossypium hirsutum* L., cv. ‘FiberMax 9058F’). Dried and ground residues of ‘Maton’ rye (*Secale cereale* L.) and ‘Lockett’ wheat (*Triticum aestivum* L.) were incorporated into Pullman clay loam soil collected from fields without recent cereal cultivation. Residues were applied at different rates representing typical field biomass levels, while the purified compound 2-benzoxazolinone (BOA) was added within ranges previously reported to affect germination and early growth in soil-amended benzoxazinoid studies [23,35].

Each pot contained 5.443 kg oven-dry soil. BOA was applied at 0, 500, or 1000 nmol g^−1^ (i.e., 0, 67.6, 135.1 mg kg^−1^), equivalent to 0, 87.8, and 175.7 kg ha^−1^ assuming homogeneous incorporation in the 0–10 cm layer.

Across the experiments, pots were arranged in a randomized complete block design with four replicates. Five cotton seeds were sown per pot. Pots were watered with a fine-mist nozzle every 1–2 days to keep the substrate moist but to minimize potential drainage. Response variables included germination, seedling height, and biomass, while leaf chlorophyll content (SPAD) was assessed using hand-held chlorophyll meter SPAD-502 (Konica Minolta, Tokyo, Japan) in experiment 3 and 4. Soil samples were also analyzed with high-performance liquid chromatography (HPLC, Shimadzu VP system, Shimadzu, Kyoto, Japan) for benzoxazinoid compounds (BOA, DIBOA, and DIMBOA) in experiments designed to capture residue decomposition and allelochemical persistence.

### 2.2. Experimental Design

We interpreted each experiment as a management scenario: Experiment 1 tested residue load as a management lever (dose–response); Experiment 2 defined a post-termination monitoring window for soil benzoxazinoid pulses; Experiment 3 evaluated carryover risk under delayed planting (long dry storage); and Experiment 4 assessed species choice (rye vs. wheat) and compound vs. residue effects (BOA vs. mixtures). This framing aligns measurements with agroecosystem functional value and resource-use efficiency.

#### 2.2.1. Experiment 1—Dose–Response of Rye and BOA on Cotton (Residue Load as a Management Lever)

Cotton was planted in soils amended with six rates of rye (0, 800, 1600, 3200, 6400, 12,800 kg ha^−1^) and three levels of BOA (0, 500 and 1000 nmol g^−1^). Germination was recorded every other day. Plant heights were measured weekly. At the end of the experiment, whole plants were collected at ground level and were dried at 60 °C to a constant weight to determine the total plant weight per treatment replicate. The experiment lasted 28 days.

#### 2.2.2. Experiment 2—Soil Residue Decomposition and Allelochemical Dynamics (Post-Termination Monitoring Window)

Soil was sampled on Days 0, 7, 14, 21, and 28. At each date, three randomly located cores (2 cm diameter, 0–10 cm depth) were collected per pot, pooled to one composite, sealed in polyethylene bags, and frozen at −20 °C until extraction. BOA, DIBOA, and DIMBOA were quantified by HPLC to track temporal dynamics.

#### 2.2.3. Experiment 3—Long-Term Persistence of Allelopathic Effects (Carryover Risk Under Delayed Planting)

To test whether allelopathic effects persisted over extended periods, pots from Experiment 2 were held dry for 283 days (>9 months) before cotton was planted again. This long fallow interval allowed assessment of residual impacts on germination, seedling growth, and chlorophyll content during a subsequent 52-day growth cycle. Measurement of cotton growth indicators was conducted as described in Experiment 1. Additionally, on Day 50 and Day 52, the chlorophyll content of the first fully expanded leaf from the top of each cotton plant was measured. The experiment was terminated 52 d after re-entering the greenhouse and 364 d after treatments had been applied.

#### 2.2.4. Experiment 4—Comparison of Rye, Wheat, and BOA (Species Choice; Compound vs. Residue)

A comparative trial of rye, wheat, and BOA was conducted in the second year using the same application rates (0, 6400, 12,800 kg ha^−1^ of rye or wheat, or 0, 500, 1000 nmol g^−1^ of BOA). The experiment lasted 52 days. Growth and chlorophyll measurements followed the same procedure as in prior experiments.

### 2.3. Soil and Chemical Analysis

Soil samples were analyzed for BOA, DIBOA, and DIMBOA using high-performance liquid chromatography (HPLC), following protocols modified from Reberg-Horton et al. [36]. Soil samples were collected at designated intervals, stored at −20 °C, and extracted with 100% methanol containing 1% acetic acid. Extracts were incubated, shaken, and centrifuged before filtration (0.22 μm pore). Filtrates were stored at −80 °C prior to analysis. Extraction was based on 13.3 mg soil equivalents per analysis. Soil extracts were analyzed by HPLC (Shimadzu LC-10 VP Series, Hypersil BDS C18, 250 × 4.0 mm, 5 μm, Shimadzu, Kyoto, Japan) using a water–methanol gradient with 20 mM acetic acid. Detection wavelengths were 255 nm (DIBOA) and 270 nm (BOA, DIMBOA). Retention times were confirmed using standards. Recovery tests were performed by spiking soils with known BOA concentrations. Extraction followed a methanol–acetic acid protocol commonly used for BXs; minor deconjugation of BX glucosides during extraction cannot be excluded [37].

### 2.4. Statistical Analysis

All plant-related data were analyzed using linear mixed-effects models (PROC MIXED, SAS 9.4; SAS Institute, Cary, NC, USA) with a randomized complete block design (four replicates). For single-time measurements, treatment was included as a fixed effect and block as a random effect. For repeated measurements, treatment, sampling date, and their interaction were specified as fixed effects, with block treated as random. Biomass data were further analyzed using ANCOVA to adjust for emergence rate as a covariate. Treatment means were separated by Tukey’s HSD test at the 5% level. Dose–response relationships were examined using linear and quadratic orthogonal contrasts. Allelochemical concentration data were log-transformed when necessary to meet assumptions of normality and were processed with PROC GLIMMIX procedure. Differences were considered significant at *p* < 0.05 unless otherwise stated. Figures were generated in Origin Pro 2025 (OriginLab Corp., Northampton, MA, USA). Error bars represent standard errors (SE) of least squares means (LS-means), which were estimated from linear mixed models using the pooled residual variance across treatments. In this approach, the error term is derived from the overall residual variance rather than from individual treatment groups, which results in comparable SE values among treatments. Consequently, the lengths of the error bars appear similar within each panel.

## 3. Results

### 3.1. Dose-Dependent Effects of Rye Residues and BOA on Cotton Germination (Experiment 1)

**Figure 1:**
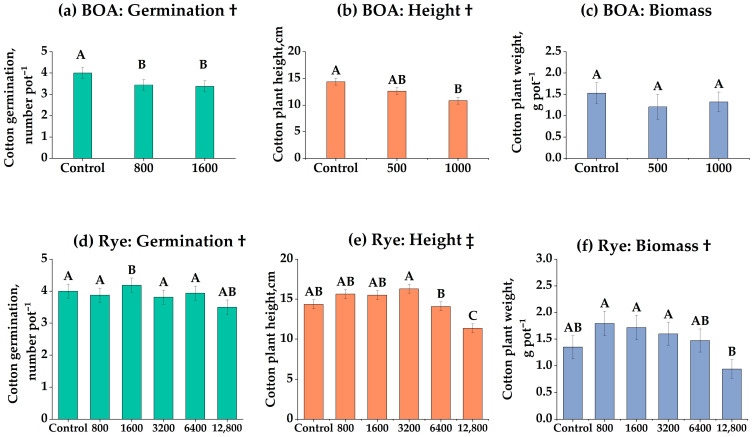
Effects of benzoxazolinone (BOA) and rye on cotton germination, plant height, and biomass. (**a**–**c**) BOA at 0, 500, and 1000 nmol g^−1^ soil; (**d**–**f**) rye at 0, 800, 1600, 3200, 6400, and 12,800 kg ha^−1^. Bars show LS-means ± SE (from mixed models). Different letters above bars indicate significant differences among treatments at *p* < 0.05 (Tukey’s HSD). † Linear trend (*p* < 0.05); ‡ Linear and quadratic trends (*p* < 0.05). Exact *p*-values for trend tests are provided in Table S1.

These dose–response functions identify a residue-biomass threshold beyond which cotton establishment proxies (germination, height, biomass) decline, quantifying the trade-off between soil cover and establishment risk (Figure 1). Cotton germination declined linearly with increasing BOA concentration (Figure 1a), with 1000 nmol g^−1^ reducing germination by 16.5% compared with the control (*p* < 0.01). In contrast, rye residues induced a hormetic response (Figure 1d): germination was enhanced at 6400 kg ha^−1^ (+7.3%, *p* < 0.05) but sharply suppressed at 12,800 kg ha^−1^ (–31%, *p* < 0.001). Plant height exhibited a similar pattern. BOA caused a linear reduction (Figure 1b; *p* < 0.05), whereas rye residues produced both linear and quadratic effects (Figure 1e), with moderate additions stimulating growth and the highest rate significantly reducing it. Biomass responded strongly to rye in a linear fashion (Figure 1f), decreasing by 45% at 12,800 kg ha^−1^ (*p* < 0.01). Under BOA, biomass was not significantly affected (Figure 1c, ns) but displayed a directional decrease, most evident at 500 nmol g^−1^, which did not reach statistical significance. These results suggest that rye residues exerted stronger and more complex influences on cotton establishment than purified BOA.

### 3.2. Decomposition of Rye Residues and BOA Dissipation in Soil (Experiment 2)

Soil benzoxazinoids varied strongly over time and showed date-specific responses to amendment rate (Table 1 and Table 2). In BOA-amended soils, BOA concentrations were dose-responsive on Day 0 and Day 7, converged across rates on Day 21 (NS), and again showed a small rate effect on Day 28 (linear). DIBOA exhibited a rate-dependent pulse only on Day 0 (linear and quadratic); at later dates it was sometimes higher in absolute terms but not responsive to BOA rate. DIMBOA was detectable and rate-responsive only on Day 7 (linear and quadratic) and otherwise absent or not rate-structured.

In rye-amended soils, BOA displayed significant linear and quadratic responses to residue rate on Day 7 and Day 21, while Day 0 and Day 28 were NS. By contrast, DIBOA and DIMBOA under rye were rate-responsive only on Day 0 (linear and quadratic); at subsequent dates these compounds were occasionally detectable but showed no significant rate effect.

Notably, DIBOA increased over time across all treatments, including the control: concentrations were very low at Day 0 but rose markedly by Day 21–28 (Table 1 and Table 2). Because rate effects for DIBOA after Day 0 were non-significant, this temporal rise appears to reflect background soil processes rather than the applied amendments. Overall, rate effects occurred only at specific sampling dates and declined rapidly thereafter, consistent with the transient nature of benzoxazinoids in soil.

### 3.3. Persistent Phytotoxicity After 9-Month Storage (Experiment 3)

**Figure 2:**
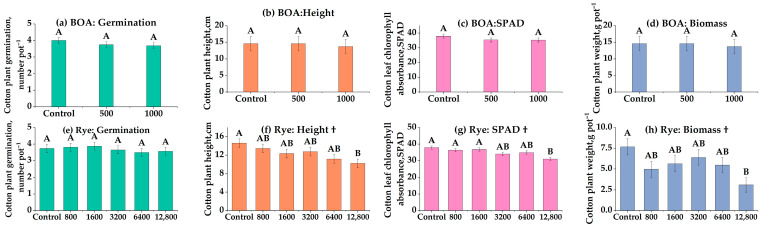
Effects of benzoxazolinone (BOA) and rye on cotton germination, plant height, chlorophyll content (SPAD), and biomass. (**a**–**d**) BOA at 0, 500, and 1000 nmol g^−1^ soil; (**e**–**h**) rye at 0, 800, 1600, 3200, 6400, and 12,800 kg ha^−1^. Bars show LS-means ± SE (from mixed models). Different letters above bars indicate significant differences among treatments at *p* < 0.05 (Tukey’s HSD). † Linear trend (*p* < 0.05). Exact *p*-values for trend tests are provided in Table S1.

Residual inhibition after long dry storage indicates carryover risk when planting is delayed, even when parent benzoxazinoids fall below detection (Figure 2). Despite the absence of detectable allelochemicals in stored soils (HPLC-negative), cotton growth remained suppressed nine months after the initial treatments (Figure 2). In BOA-amended soils, no significant effects were observed on germination, height, SPAD, or biomass (Figure 2a–d, ns). Nevertheless, a decreasing trend in biomass and SPAD was evident, particularly at the intermediate rate (500 nmol g^−1^ soil), which, although not statistically significant, suggests a potential inhibitory tendency. By contrast, rye residues exerted pronounced residual effects. Germination was unaffected (Figure 2e, ns), but plant height and SPAD decreased linearly with residue rate (Figure 2f,g; *p* < 0.05), and biomass declined strongly in a linear manner, with a 45% reduction at 12,800 kg ha^−1^ (Figure 2h; *p* < 0.001). These findings demonstrate that long-term suppression of cotton growth was not attributable to BOA alone, but rather to a more complex mixture of rye-derived allelochemicals or their degradation products that persisted in the soil. This suggests that detectable BOA levels are not always required for phytotoxicity to occur, reinforcing the importance of degradation products and cumulative residue effects, which are further elaborated in the Discussion section.

### 3.4. Comparative Phytotoxicity of Rye, Wheat, and BOA (Experiment 4)

**Figure 3:**
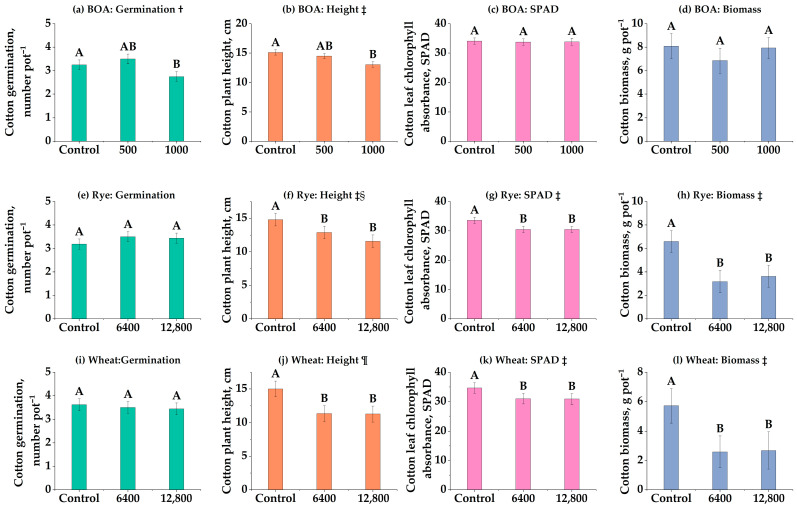
Effects of benzoxazolinone (BOA), rye, and wheat on cotton germination, plant height, chlorophyll content (SPAD), and biomass. (**a**–**d**) BOA at 0, 500, and 1000 nmol g^−1^ soil; (**e**–**h**) rye at 0, 6400, and 12,800 kg ha^−1^; (**i**–**l**) wheat at 0, 6400, and 12,800 kg ha^−1^. Bars show LS-means ± SE (from mixed models). Different letters above bars indicate significant differences among treatments at *p* < 0.05 (Tukey’s HSD). † Quadratic trend (*p* < 0.05); ‡ Linear trend (*p* < 0.05); § Date × rate interaction (*p* < 0.01, magnitude only), ¶ Linear (*p* < 0.05) and quadratic (*p* < 0.05) trend. Exact *p*-values for trend tests are provided in Table S1.

Direct comparisons of species and BOA clarify species choice as a practical lever: rye > wheat > BOA in inhibitory strength, with trait-specific exceptions (Figure 3). Cotton germination exhibited a quadratic decline under BOA (Figure 3a), while wheat and rye residues showed no significant effects (Figure 3e,i; ns). Plant height was consistently suppressed across all three inputs, with rye and wheat exerting stronger inhibitory effects than BOA (Figure 3b,f,j). Chlorophyll content (SPAD) followed a similar trend: BOA had no significant impact (Figure 3c), whereas both rye and wheat significantly reduced chlorophyll levels (Figure 3g,k; *p* < 0.05). Biomass was most strongly reduced by rye, followed by wheat, while BOA again showed no significant effect (Figure 3d,h,l); at 12,800 kg ha^−1^, rye reduced biomass by 36.5% (*p* < 0.01) and wheat by 23.7%. Collectively, these results demonstrate that both purified BOA and cereal residues can suppress cotton growth, but rye residues exert the most potent and consistent phytotoxic effects under controlled conditions.

## 4. Discussion

Framing these results as functional trade-offs links benzoxazinoid pulses and persistence to decision-relevant levers that enhance establishment reliability without sacrificing soil cover services. This series of greenhouse experiments demonstrated that both rye residues and purified BOA suppressed cotton germination and early growth in a dose-dependent manner, with rye exerting stronger and more persistent effects than BOA alone (Experiment 1; Figure 1a–f). These findings are consistent with field observations that rye cover crops can negatively affect subsequent cotton yields [26,27]. The hormetic stimulation at moderate rye biomass (6400 kg ha^−1^) followed by strong inhibition at higher levels (Experiment 1; Figure 1d–f) mirrors reports from other allelopathy studies, where intermediate doses of benzoxazinoids sometimes stimulate growth before inhibition dominates [38,39]. Linear and quadratic contrasts confirmed that rye exerted a nonlinear dose–response, while BOA followed a largely linear inhibitory trend (Experiment 1; Figure 1a–c).

The dissipation experiments (Experiment 2) highlighted rapid degradation of BOA and its derivatives, with detectable levels declining within weeks (Table 1 and Table 2). This aligns with reports that benzoxazinoids, including BOA, DIBOA, and DIMBOA, are unstable in soil, with half-lives of hours to days depending on microbial activity and environmental conditions [23,40,41]. In our data, rate effects were confined to specific sampling dates (Table 1 and Table 2): in BOA-amended soils, BOA responded to dose on Day 0, Day 7, and Day 28 (Day 21, NS), DIBOA responded only on Day 0 (L, Q), and DIMBOA responded only on Day 7 (L, Q); in rye-amended soils, BOA showed dose responses on Day 7 and Day 21, whereas DIBOA and DIMBOA responded only on Day 0. Nevertheless, detectable background levels in control soils support the notion that benzoxazinoid signatures can persist long after small grains have been grown in a field, consistent with observations by Silva et al. [7] and Krogh et al. [42]. Importantly, although BOA degraded rapidly, rye amendments sustained inhibitory effects on cotton even after nine months of dry soil storage (Experiment 3). This suggests that complex mixtures of degradation products—such as 2-amino-3H-phenoxazin-3-one (APO), 2-hydroxy-1,4-benzoxazin-3-one (HBOA), and 2-hydroxy-7-methoxy-1,4-benzoxazin-3-one (HMBOA)—may contribute to persistent phytotoxicity [43,44], corroborating findings from Schulz et al. [18] and others that allelochemical activity often results from transformation products rather than parent compounds alone [35,45]. Empirical evidence from Rice et al. [23] further shows that HMBOA-glc and HMBOA dominate immediately after rye termination, followed by rapid decay—highlighting a transient yet potent allelochemical pulse from residues.

We also found that benzoxazinoids were rate-significant only at specific dates whereas compounds could remain detectable at other dates without dose responsiveness (Experiment 2; Table 1 and Table 2). Notably, DIBOA increased over time across all treatments, including controls, indicating a time-dependent rise that was not driven by amendment rate after Day 0. This pattern is consistent with mobilization of background or bound benzoxazinoid pools and/or transformation among benzoxazinoid forms, with short-lived intermediates (e.g., DIMBOA) degrading or rearranging while DIBOA accumulates [32,43]. Such episodic and compound-specific dynamics emphasize the importance of sampling frequency and timing in allelopathy studies, as rate-dependent peaks may be missed if measurements are not synchronized with these transient processes.

The late-season increase in DIBOA—even in controls—likely reflects mobilization of background or bound benzoxazinoid pools rather than continued inputs from our treatments. Legacy BX glycosides from past grasses or humus-bound residues can be enzymatically (β-glucosidase) or abiotically deconjugated over weeks, releasing DIBOA, whereas DIMBOA is less stable and rearranges or degrades more rapidly, and BOA is quickly transformed to phenoxazinones (e.g., APO). A minor contribution from extraction-induced deconjugation under methanol/weak-acid extraction cannot be excluded. The absence of significant rate effects on DIBOA after Day 0 supports the view that time-dependent soil processes dominate later DIBOA levels rather than treatment dose [23,46,47,48,49] (Experiment 2; Table 1 and Table 2).

In our direct comparison (Experiment 4), the rye cultivar tested showed stronger inhibition than the wheat cultivar, while BOA alone was weaker (Figure 3). This result is consistent with reports that cereals differ in allelopathic potential [50,51,52], though differences can depend on cultivar choice and experimental conditions rather than a universal superiority of rye over wheat. The SPAD data further indicate reduced chlorophyll at high rye and wheat residue loads, consistent with allelopathy-induced impairment of photosynthesis [53,54]. Similar reductions have been observed in cotton grown in small-grain stubble under field conditions [26], linking physiological responses to field-scale yield penalties.

The consistency of rye-driven suppression across short-term (28 d), intermediate (51 d), and long-term (364 d) experiments underscores the resilience of allelopathic effects (Experiment 1: Figure 1; Experiment 4: Figure 3; Experiment 3: Figure 2). Our findings align with Yenish et al. [55], who reported that DIBOA and related compounds persisted for several months in rye residues under field conditions, and with Li et al. [26,27], who observed measurable suppression of cotton more than a year after rye termination. Together, these studies support our observation that residue-mediated inhibition can persist even when parent BOA is no longer measurable in soil, highlighting the complexity of benzoxazinoid degradation pathways.

While some studies attribute rapid dissipation to microbial mineralization [42,56], others suggest that degradation intermediates can retain or even enhance bioactivity [42,57]. Beyond degradation dynamics, benzoxazinoids also exhibit broad ecological functions, influencing defense responses, nutrient uptake, and even developmental traits such as flowering time [58]. These multi-functional roles help explain why residue mixtures can produce richer and more persistent biological responses than BOA alone.

From a management perspective, the intensity of cotton suppression is strongly linked to rye residue biomass. In long-term grazing–cover crop systems, grazing reduces residue loads and mitigates negative effects on subsequent cotton [27]. Our greenhouse results reinforce this interpretation: stronger inhibition at higher rye doses parallels biomass-dependent responses observed in grazed versus ungrazed plots. Thus, residue management strategies—such as grazing, mechanical removal, or selecting less-allelopathic cultivars—can help balance soil conservation benefits with the risk of reduced cotton performance.

Allelopathy is unlikely to be the sole pathway by which cover crops influence subsequent crops. In field conditions, changes in nutrient cycling, moisture dynamics, and microbial communities interact with allelochemical effects [56,59]. For example, benzoxazinoid degradation can be accelerated by specific soil microbes (e.g., *Pseudomonas* spp., *Bacillus* spp., *Paenibacillus polymyxa*) that mineralize or detoxify allelochemicals [40,42]. Soil pH, texture, and organic matter also modulate persistence and activity [41,60]. In our controlled greenhouse conditions, where fertility and moisture were non-limiting, suppression can be attributed primarily to allelochemical activity—indicating that even under optimal conditions, benzoxazinoid-mediated allelopathy can substantially impair cotton establishment.

At the agroecosystem scale, residue biomass and planting timing are controllable levers that translate biochemical insight into functional gains. High rye residue enhances soil cover and weed suppression but can cross an allelopathic threshold that compromises cotton establishment; moderate loads balance services and risks. The early post-termination window (first 1–3 weeks) is critical for monitoring benzoxazinoid pulses and guiding termination-to-planting intervals. Species choice further tunes risk: rye is generally more suppressive than wheat, while BOA alone shows weaker, trait-specific effects—underscoring the complexity of residue-derived mixtures and transformation products. Operationally, grazing or partial residue removal, delaying planting until the pulse wanes, and deploying cultivars with higher tolerance can improve resource-use efficiency without sacrificing soil conservation services. These system level adjustments reconcile soil health objectives (erosion control, carbon inputs, weed suppression) with reliable establishment and early-season productivity in cover crop–cotton systems.

Taken together, these greenhouse experiments provide strong evidence that allelopathic interactions from rye and wheat residues—mediated by benzoxazinoids and their degradation products—can suppress cotton establishment in a dose-dependent and persistent manner. While cover crops are central to soil conservation and sustainable intensification, their management must be calibrated to avoid unintended yield penalties in cotton-based systems. Integrating biochemical insights with field-scale agronomy—such as optimized residue biomass, cultivar selection, and, where appropriate, microbial inoculation—offers practical avenues to capture the benefits of cover crops while minimizing risk. In practice, keeping rye residue below thresholds that trigger carryover inhibition—via grazing, partial removal, or choice of less-allelopathic cultivars—should be prioritized where rapid cotton stand establishment is critical.

Greenhouse conditions in this study minimized water and nutrient constraints and can therefore amplify allelopathic signals relative to on-farm settings. In producer fields, moisture deficits and residue-induced nitrogen immobilization frequently co-occur and can confound or mask allelopathic effects, shifting early stress toward resource competition under high-biomass rye mulches [61,62,63]. Moreover, the fate and activity of benzoxazinoids are microbially mediated; soil and microcosm studies report rapid BOA/DIMBOA turnover and frequent predominance of APO in soils, indicating strong microbial processing that depends on moisture and temperature regimes [35,52]. Management further modulates field outcomes: termination timing and residue load alter microclimate, pathogen pressure, and competition, with multi-location trials showing limited yield penalties under timely termination but higher risk when mulch biomass is high and planting is delayed [64,65]. Taken together, our greenhouse estimates should be viewed as a biochemical upper bound; field validation across moisture and fertility gradients is needed to partition allelopathy vs. competition and to calibrate management thresholds.

Allelopathic impacts of cover crop species and termination timing on cotton germination and seedling growth [22,51,66] and cotton cultivars vary in tolerance to benzoxazinoids [67], screening/identifying tolerant cotton germplasm is a practical route to integrate cover crops without sacrificing yield. In parallel, resolving BOA transformation pathways and the roles of secondary metabolites such as APO [19,68]—using advanced metabolomics and microbiome analyses—will clarify why residue-derived effects can persist beyond parent-compound detection. Looking forward, research should validate these greenhouse findings across environments, resolve the transformation pathways and persistence of benzoxazinoid derivatives in soil, and identify cotton germplasm with greater allelopathic tolerance. These steps are essential to balance the ecological services of cereal cover crops with the productivity requirements of cotton-based systems, thereby supporting sustainable crop rotations. Ultimately, integrating allelopathy knowledge into crop management will advance climate-resilient agriculture and align with global Sustainable Development Goals on sustainable production and environmental stewardship.

## 5. Conclusions

This study shows that rye and wheat residues and the purified allelochemical benzoxazolinone (BOA) suppress cotton establishment in a dose-dependent, trait-specific manner. Rye exerted the strongest and most persistent inhibition, wheat showed intermediate effects, and BOA was weaker—affecting germination and height but generally not biomass or SPAD. Despite rapid BOA dissipation in soil, rye-driven suppression persisted after prolonged dry storage, implicating transformation products and soil biotic interactions. From a management perspective, excessive rye biomass amplified phytotoxicity, whereas moderate loads occasionally stimulated early growth (hormesis). Framed at the agroecosystem scale, keeping residue loads below inhibitory thresholds and adjusting termination-to-planting intervals to avoid early benzoxazinoid pulses provide practical levers to retain cover crop services (soil cover and weed suppression) while minimizing carryover risks to cotton. Species and cultivar choice further tunes risk. These actions improve resource use efficiency and support resilient, sustainable cereal–cotton systems, offering decision-relevant guidance for farmers and advisers.

## Figures and Tables

**Table 1:** Soil concentrations of benzoxazolinone (BOA) and its derivatives (mg kg^−1^) following amendment with BOA at 0 (control), 500, or 1000 nmol g^−1^ soil in Experiment 2.

Allelochemical	Date	Control	500 BOA	1000 BOA	Trend *
BOA	Day 0	0.053	5.06	99.02	L
	Day 7	0.13	0.75	0.86	L
	Day 21	0.66	0.66	0.7	NS
	Day 28	0.024	0.033	0.42	L
DIBOA	Day 0	0.0016	1.99	8.95	L, Q
	Day 7	0.0016	0.0041	0	NS
	Day 21	4.04	4.29	1.72	NS
	Day 28	3.81	3.45	5.59	NS
DIMBOA	Day 0	0	0	0	NS
	Day 7	0	0	0.066	L, Q
	Day 21	0	0	0	NS
	Day 28	0	0	0	NS

* L indicates a significant linear trend (*p* < 0.05); Q indicates a significant quadratic trend (*p* < 0.05); NS indicates not significant.

**Table 2:** Soil concentrations of BOA and its derivatives (mg kg^−1^) following amendment with rye residues at 0 (control), 800, 1600, 3200, 6400, or 12,800 kg ha^−1^ in Experiment 2.

Allelochemical	Date	Control	800	1600	3200	6400	12,800	Trend *
BOA	Day 0	0.05	0.018	0.022	0.061	0.062	0.12	NS
	Day 7	0.13	0.046	0.21	0.081	0.023	0.019	L, Q
	Day 21	0.63	0.31	0.22	0.092	0.061	0.051	L, Q
	Day 28	0.02	0.042	0.025	0.021	0.051	0.058	NS
DIBOA	Day 0	0.0016	0.0016	0.0041	0	0.18	0.067	L, Q
	Day 7	0.0016	0.013	0.0017	0.0017	0.015	0.015	NS
	Day 21	4.04	0.21	0.52	4.16	1.02	1.26	NS
	Day 28	3.79	5.99	1.88	4.56	3.67	0.11	NS
DIMBOA	Day 0	0	0	0	0	0.77	0.95	L, Q
	Day 7	0	0	0	0	0	0.009	NS
	Day 21	0	0	0	0.007	0	0.009	NS
	Day 28	0	0.007	0.02	0.007	0.019	0.037	NS

* L indicates a significant linear trend (*p* < 0.05); Q indicates a significant quadratic trend (*p* < 0.05); NS indicates not significant.

## Data Availability

All data and supporting information are openly available at Zenodo (https://doi.org/10.5281/zenodo.16967252).

## Supplementary Materials

The following supporting information can be downloaded at: https://www.mdpi.com/article/10.3390/biology14101321/s1, Table S1: Mixed-model tests for linear (L) and quadratic (Q) dose–response trends by experiment, material, and trait.

## Abbreviations

The following abbreviations are used in this manuscript:

APO	2-Amino-3H-phenoxazin-3-one
BOA	2-Benzoxazolinone
DIBOA	2,4-Dihydroxy-1,4-benzoxazin-3-one
DIMBOA	2,4-Dihydroxy-7-methoxy-1,4-benzoxazin-3-one
HBOA	2-Hydroxy-1,4-benzoxazin-3-one
HMBOA	2-Hydroxy-7-methoxy-1,4-benzoxazin-3-one

## References

[1] Quintarelli V., Radicetti E., Allevato E., Stazi S.R., Haider G., Abideen Z., Bibi S., Jamal A., Mancinelli R. (2022). Cover Crops for Sustainable Cropping Systems: A Review. Agriculture.

[2] Gazoulis I., Kanatas P., Petraki D., Antonopoulos N., Kokkini M., Danaskos M., Travlos I. (2025). Enhancing Agroecosystem Sustainability by Means of Cover Crops in the Era of Climate Change. Agronomy.

[3] Blanco-Canqui H., Ruis S.J., Holman J.D., Creech C.F., Obour A.K. (2022). Can Cover Crops Improve Soil Ecosystem Services in Water-Limited Environments? A Review. Soil Sci. Soc. Am. J..

[4] Burke J.A., Lewis K.L., DeLaune P.B., Cobos C.J., Keeling J.W. (2022). Soil Water Dynamics and Cotton Production Following Cover Crop Use in a Semi-Arid Ecoregion. Agronomy.

[5] Toler H.D., Augé R.M., Benelli V., Allen F.L., Ashworth A.J. (2019). Global Meta-Analysis of Cotton Yield and Weed Suppression from Cover Crops. Crop Sci..

[6] Yousefi M., Dray A., Ghazoul J. (2024). Assessing the Effectiveness of Cover Crops on Ecosystem Services: A Review of the Benefits, Challenges, and Trade-Offs. Int. J. Agric. Sustain..

[7] Camargo Silva G., Bagavathiannan M. (2023). Mechanisms of Weed Suppression by Cereal Rye Cover Crop: A Review. Agron. J..

[8] Adhikari L., Mohseni-Moghadam M., Missaoui A. (2018). Allelopathic Effects of Cereal Rye on Weed Suppression and Forage Yield in Alfalfa. Am. J. Plant Sci..

[9] Rebong D., Henriquez Inoa S., Moore V.M., Reberg-Horton S.C., Mirsky S., Murphy J.P., Leon R.G. (2024). Breeding Allelopathy in Cereal Rye for Weed Suppression. Weed Sci..

[10] Ullah H., Khan N., Khan I.A. (2023). Complementing Cultural Weed Control with Plant Allelopathy: Implications for Improved Weed Management in Wheat Crop. Acta Ecol. Sin..

[11] van der Meulen A., Chauhan B.S. (2017). A Review of Weed Management in Wheat Using Crop Competition. Crop Prot..

[12] Marcos F.M., Acharya J., Parvej M.d.R., Robertson A.E., Licht M.A. (2023). Cereal Rye Cover Crop Seeding Method, Seeding Rate, and Termination Timing Effects Corn Development and Seedling Disease. Agron. J..

[13] Koehler-Cole K., Everhart S.E., Gu Y., Proctor C.A., Marroquin-Guzman M., Redfearn D.D., Elmore R.W. (2020). Is Allelopathy from Winter Cover Crops Affecting Row Crops?. Agric. Environ. Lett..

[14] Almeida T.F., Robinson E., Matthiesen-Anderson R., Robertson A.E., Basche A. (2024). Effect of Cover Crop Species and Termination Timing on Corn Growth and Seedling Disease. Agron. J..

[15] Schandry N., Becker C. (2020). Allelopathic Plants: Models for Studying Plant–Interkingdom Interactions. Trends Plant Sci..

[16] Anastasi U., Scavo A. (2023). Cropping Systems and Agronomic Management Practices of Field Crops. Agronomy.

[17] Hickman D.T., Comont D., Rasmussen A., Birkett M.A. (2023). Novel and Holistic Approaches Are Required to Realize Allelopathic Potential for Weed Management. Ecol. Evol..

[18] Schulz M., Marocco A., Tabaglio V., Macias F.A., Molinillo J.M.G. (2013). Benzoxazinoids in Rye Allelopathy—From Discovery to Application in Sustainable Weed Control and Organic Farming. J. Chem. Ecol..

[19] Kudjordjie E.N., Sapkota R., Steffensen S.K., Fomsgaard I.S., Nicolaisen M. (2019). Maize Synthesized Benzoxazinoids Affect the Host Associated Microbiome. Microbiome.

[20] Ozaki Y., Kato-Noguchi H. (2015). Effects of Benzoxazinoids in Wheat Residues May Inhibit the Germination, Growth and Gibberellin-Induced α-Amylase Activity in Rice. Acta Physiol. Plant.

[21] Laschke L., Schütz V., Schackow O., Sicker D., Hennig L., Hofmann D., Dörmann P., Schulz M. (2022). Survival of Plants During Short-Term BOA-OH Exposure: ROS Related Gene Expression and Detoxification Reactions Are Accompanied With Fast Membrane Lipid Repair in Root Tips. J. Chem. Ecol..

[22] Scavo A., Pandino G., Restuccia A., Caruso P., Lombardo S., Mauromicale G. (2022). Allelopathy in Durum Wheat Landraces as Affected by Genotype and Plant Part. Plants.

[23] Rice C.P., Otte B.A., Kramer M., Schomberg H.H., Mirsky S.B., Tully K.L. (2022). Benzoxazinoids in Roots and Shoots of Cereal Rye (*Secale cereale*) and Their Fates in Soil after Cover Crop Termination. Chemoecology.

[24] Lewis K.L., Burke J.A., Keeling W.S., McCallister D.M., DeLaune P.B., Keeling J.W. (2018). Soil Benefits and Yield Limitations of Cover Crop Use in Texas High Plains Cotton. Agron. J..

[25] Allen V.G., Baker M.T., Segarra E., Brown C.P. (2007). Integrated Irrigated Crop–Livestock Systems in Dry Climates. Agron. J..

[26] Li Y., Allen V.G., Chen J., Hou F., Brown C.P., Green P. (2013). Allelopathic Influence of a Wheat or Rye Cover Crop on Growth and Yield of No-Till Cotton. Agron. J..

[27] Li Y., Allen V.G., Hou F., Chen J., Brown C.P. (2013). Steers Grazing a Rye Cover Crop Influence Growth of Rye and No-Till Cotton. Agron. J..

[28] Chinchilla N., Marín D., Oliveros-Bastidas A., Molinillo J.M.G., Macías F.A. (2015). Soil Biodegradation of a Benzoxazinone Analog Proposed as a Natural Products-Based Herbicide. Plant Soil.

[29] Hu L., Robert C.A.M., Cadot S., Zhang X., Ye M., Li B., Manzo D., Chervet N., Steinger T., van der Heijden M.G.A. (2018). Root Exudate Metabolites Drive Plant-Soil Feedbacks on Growth and Defense by Shaping the Rhizosphere Microbiota. Nat. Commun..

[30] Xiao H., van Es H.M., Chen Y., Wang B., Zhao Y., Sui P. (2022). Crop Rotational Diversity Influences Wheat–Maize Production Through Soil Legacy Effects in the North China Plain. Int. J. Plant Prod..

[31] Lou Y., Davis A.S., Yannarell A.C. (2016). Interactions between Allelochemicals and the Microbial Community Affect Weed Suppression Following Cover Crop Residue Incorporation into Soil. Plant Soil.

[32] Fadiji A.E., Adeniji A., Lanrewaju A.A., Babalola O.O. (2025). Dynamics of Soil Microbiome and Allelochemical Interactions: An Overview of Current Knowledge and Prospects. Ann. Microbiol..

[33] Belz R.G., Duke S.O. (2022). Modelling Biphasic Hormetic Dose Responses to Predict Sub-NOAEL Effects Using Plant Biology as an Example. Curr. Opin. Toxicol..

[34] Kostina-Bednarz M., Płonka J., Barchanska H. (2023). Allelopathy as a Source of Bioherbicides: Challenges and Prospects for Sustainable Agriculture. Rev. Environ. Sci. Biotechnol..

[35] Schütz V., Frindte K., Cui J., Zhang P., Hacquard S., Schulze-Lefert P., Knief C., Schulz M., Dörmann P. (2021). Differential Impact of Plant Secondary Metabolites on the Soil Microbiota. Front. Microbiol..

[36] Reberg-Horton S.C., Burton J.D., Danehower D.A., Ma G., Monks D.W., Murphy J.P., Ranells N.N., Williamson J.D., Creamer N.G. (2005). Changes Over Time in the Allelochemical Content of Ten Cultivars of Rye (*Secale cereale* L.). J. Chem. Ecol..

[37] Niculaes C., Abramov A., Hannemann L., Frey M. (2018). Plant Protection by Benzoxazinoids—Recent Insights into Biosynthesis and Function. Agronomy.

[38] Hickman D.T., Rasmussen A., Ritz K., Birkett M.A., Neve P. (2021). Review: Allelochemicals as Multi-kingdom Plant Defence Compounds: Towards an Integrated Approach. Pest Manag. Sci..

[39] Abbas T., Nadeem M.A., Tanveer A., Chauhan B.S. (2017). Can Hormesis of Plant-Released Phytotoxins Be Used to Boost and Sustain Crop Production?. Crop Prot..

[40] Fomsgaard I.S., Mortensen A.G., Carlsen S.C.K. (2004). Microbial Transformation Products of Benzoxazolinone and Benzoxazinone Allelochemicals–A Review. Chemosphere.

[41] Scavo A., Abbate C., Mauromicale G. (2019). Plant Allelochemicals: Agronomic, Nutritional and Ecological Relevance in the Soil System. Plant Soil.

[42] Schütz V., Bigler L., Girel S., Laschke L., Sicker D., Schulz M. (2019). Conversions of Benzoxazinoids and Downstream Metabolites by Soil Microorganisms. Front. Ecol. Evol..

[43] Mwendwa J.M., Weston P.A., Weidenhamer J.D., Fomsgaard I.S., Wu H., Gurusinghe S., Weston L.A. (2021). Metabolic Profiling of Benzoxazinoids in the Roots and Rhizosphere of Commercial Winter Wheat Genotypes. Plant Soil.

[44] Wouters F.C., Gershenzon J., Vassão D.G. (2016). Benzoxazinoids: Reactivity and Modes of Action of a Versatile Class of Plant Chemical Defenses. J. Braz. Chem. Soc..

[45] Singh A.A., Rajeswari G., Nirmal L.A., Jacob S. (2021). Synthesis and Extraction Routes of Allelochemicals from Plants and Microbes: A Review. Rev. Anal. Chem..

[46] Macías F.A., Oliveros-Bastidas A., Marín D., Castellano D., Simonet A.M., Molinillo J.M.G. (2004). Degradation Studies on Benzoxazinoids. Soil Degradation Dynamics of 2,4-Dihydroxy-7-Methoxy-(2H)-1,4-Benzoxazin-3(4H)-One (DIMBOA) and Its Degradation Products, Phytotoxic Allelochemicals from Gramineae. J. Agric. Food Chem..

[47] Understrup A.G., Ravnskov S., Hansen H.C.B., Fomsgaard I.S. (2005). Biotransformation of 2-Benzoxazolinone to 2-Amino-(3H)-Phenoxazin-3-One and 2-Acetylamino-(3H)-Phenoxazin-3-One in Soil. J. Chem. Ecol..

[48] Krogh S.S., Mensz S.J.M., Nielsen S.T., Mortensen A.G., Christophersen C., Fomsgaard I.S. (2006). Fate of Benzoxazinone Allelochemicals in Soil after Incorporation of Wheat and Rye Sprouts. J. Agric. Food Chem..

[49] Morant A.V., Jørgensen K., Jørgensen C., Paquette S.M., Sánchez-Pérez R., Møller B.L., Bak S. (2008). β-Glucosidases as Detonators of Plant Chemical Defense. Phytochemistry.

[50] González-García E., Sánchez-Moreiras A.M., Vieites-Álvarez Y. (2025). Allelopathic Potential and Chemical Profile of Wheat, Rice and Barley against the Herbicide-Resistant Weeds Portulaca Oleracea L. and Lolium Rigidum Gaud. BMC Plant Biol..

[51] Worthington M., Reberg-Horton C. (2013). Breeding Cereal Crops for Enhanced Weed Suppression: Optimizing Allelopathy and Competitive Ability. J. Chem. Ecol..

[52] Reiss A., Fomsgaard I.S., Mathiassen S.K., Kudsk P. (2018). Weed Suppressive Traits of Winter Cereals: Allelopathy and Competition. Biochem. Syst. Ecol..

[53] Siyar S., Majeed A., Muhammad Z., Ali H., Inayat N. (2019). Allelopathic Effect of Aqueous Extracts of Three Weed Species on the Growth and Leaf Chlorophyll Content of Bread Wheat. Acta Ecol. Sin..

[54] Hussain M.I., Reigosa M.J. (2021). Secondary Metabolites, Ferulic Acid and p-Hydroxybenzoic Acid Induced Toxic Effects on Photosynthetic Process in *Rumex acetosa* L.. Biomolecules.

[55] Yenish J.P., Worsham A.D., Chilton W.S. (1995). Disappearance of DIBOA-Glucoside, DIBOA, and BOA from Rye (*Secale cereale* L.) Cover Crop Residue. Weed Sci..

[56] Weston P.A., Parvin S., Hendriks P.-W., Gurusinghe S., Rebetzke G.J., Weston L.A. (2025). Impact of Year and Genotype on Benzoxazinoids and Their Microbial Metabolites in the Rhizosphere of Early-Vigour Wheat Genotypes in Southern Australia. Plants.

[57] Hussain M.I., Araniti F., Schulz M., Baerson S., Vieites-Álvarez Y., Rempelos L., Bilsborrow P., Chinchilla N., Macías F.A., Weston L.A. (2022). Benzoxazinoids in Wheat Allelopathy—From Discovery to Application for Sustainable Weed Management. Environ. Exp. Bot..

[58] Zhou S., Richter A., Jander G. (2018). Beyond Defense: Multiple Functions of Benzoxazinoids in Maize Metabolism. Plant Cell Physiol..

[59] Zambelli A., Nocito F.F., Araniti F. (2025). Unveiling the Multifaceted Roles of Root Exudates: Chemical Interactions, Allelopathy, and Agricultural Applications. Agronomy.

[60] Geddes C.M., Gulden R.H. (2021). Wheat and Cereal Rye Inter-Row Living Mulches Interfere with Early Season Weeds in Soybean. Plants.

[61] Won S., Rejesus R.M., Poncet A.M., Aglasan S., Thapa R., Tulley K.L., Reberg-Horton C., Cabrera M.L., Davis B.W., Gaskin J. (2024). Understanding the Yield Impacts of Alternative Cover Crop Families and Mixtures: Evidence from Side-by-Side Plot-Level Panel Data. Agrosyst. Geosci. Environ..

[62] Abdalla M., Hastings A., Cheng K., Yue Q., Chadwick D., Espenberg M., Truu J., Rees R.M., Smith P. (2019). A Critical Review of the Impacts of Cover Crops on Nitrogen Leaching, Net Greenhouse Gas Balance and Crop Productivity. Glob. Change Biol..

[63] Fernandez C., Monnier Y., Santonja M., Gallet C., Weston L.A., Prévosto B., Saunier A., Baldy V., Bousquet-Mélou A. (2016). The Impact of Competition and Allelopathy on the Trade-Off between Plant Defense and Growth in Two Contrasting Tree Species. Front. Plant Sci..

[64] Godar A.S., Norsworthy J.K., Barber L.T. (2024). Effect of Cereal Rye Cover Crop Termination Timings on Weed Control and Corn Yield under a Two-Pass Herbicide Program. Front. Agron..

[65] Silva T.S., Mourtzinis S., McMechan A.J., Carmona G.I., Potter B.D., Tilmon K.J., Hesler L.S., Seiter N.J., Wright R., Osborne S.L. (2024). Cereal Rye Cover Crop Termination at or before Soybean Planting Has Minimal Effect on Soybean Yield across the Midwestern US. Field Crops Res..

[66] Bertholdsson N.-O. (2010). Breeding Spring Wheat for Improved Allelopathic Potential. Weed Res..

[67] Shekoofa A., Safikhan S., Raper T.B., Butler S.A. (2020). Allelopathic Impacts of Cover Crop Species and Termination Timing on Cotton Germination and Seedling Growth. Agronomy.

[68] Macías F.A., Mejías F.J., Molinillo J.M. (2019). Recent Advances in Allelopathy for Weed Control: From Knowledge to Applications. Pest Manag. Sci..

